# Survival of itinerant excitations and quantum spin state transitions in YbMgGaO_4_ with chemical disorder

**DOI:** 10.1038/s41467-021-25247-6

**Published:** 2021-08-16

**Authors:** X. Rao, G. Hussain, Q. Huang, W. J. Chu, N. Li, X. Zhao, Z. Dun, E. S. Choi, T. Asaba, L. Chen, L. Li, X. Y. Yue, N. N. Wang, J.-G. Cheng, Y. H. Gao, Y. Shen, J. Zhao, G. Chen, H. D. Zhou, X. F. Sun

**Affiliations:** 1 Hefei National Laboratory for Physical Sciences at Microscale, Department of Physics, and Key Laboratory of Strongly-Coupled Quantum Matter Physics (CAS), University of Science and Technology of China, Hefei, Anhui People’s Republic of China; 2 Department of Physics and Astronomy, University of Tennessee, Knoxville, TN USA; 3 School of Physical Sciences, University of Science and Technology of China, Hefei, Anhui People’s Republic of China; 4 National High Magnetic Field Laboratory, Florida State University, Tallahassee, FL USA; 5 Department of Physics, University of Michigan, Ann Arbor, MI USA; 6 Institute of Physical Science and Information Technology, Anhui University, Hefei, Anhui People’s Republic of China; 7 Beijing National Laboratory for Condensed Matter Physics and Institute of Physics, Chinese Academy of Sciences, Beijing, People’s Republic of China; 8 State Key Laboratory of Surface Physics and Department of Physics, Fudan University, Shanghai, People’s Republic of China; 9 Department of Physics and HKU-UCAS Joint Institute for Theoretical and Computational Physics at Hong Kong, The University of Hong Kong, Hong Kong, China

**Keywords:** Magnetic properties and materials, Magnetic properties and materials

## Abstract

A recent focus of quantum spin liquid (QSL) studies is how disorder/randomness in a QSL candidate affects its true magnetic ground state. The ultimate question is whether the QSL survives disorder or the disorder leads to a “spin-liquid-like” state, such as the proposed random-singlet (RS) state. Since disorder is a standard feature of most QSL candidates, this question represents a major challenge for QSL candidates. YbMgGaO_4_, a triangular lattice antiferromagnet with effective spin-1/2 Yb^3+^ions, is an ideal system to address this question, since it shows no long-range magnetic ordering with Mg/Ga site disorder. Despite the intensive study, it remains unresolved as to whether YbMgGaO_4_ is a QSL or in the RS state. Here, through ultralow-temperature thermal conductivity and magnetic torque measurements, plus specific heat and DC magnetization data, we observed a residual *κ*_0_/*T* term and series of quantum spin state transitions in the zero temperature limit for YbMgGaO_4_. These observations strongly suggest that a QSL state with itinerant excitations and quantum spin fluctuations survives disorder in YbMgGaO_4_.

## Abstract (web summary)

It remains an open question as to whether the quantum spin liquid state survives material disorder, or is replaced by some spin-liquid like state. Here, Rao et al succeed in resolving a resolving a *κ*_0_/T residual in the thermal conductivity of YbMgGaO_4_ strongly suggesting the survival of the quantum spin liquid state.

## Introduction

A quantum spin liquid (QSL) is an exotic quantum state in which spins are highly entangled and remain disordered down to zero-temperature limit^1–4^. It has attracted intensive interest because of its potential relevance to high-temperature superconductivity and quantum information applications. The most fascinating feature of certain gapless QSL is that its fermionic-like spin excitations, or spinons, behave like mobile charge carriers in a paramagnetic metal with a Fermi surface^1–4^. Since the first reported gapless QSL candidate EtMe_3_Sb[Pd(dmit)_2_]_2_^5^, a spin-1/2 triangular lattice antiferromagnet (TAF) in 2010, the search for other candidates remains as a hot topic during the last decade. One commonly used experimental probe for QSL is a broad continuous magnetic excitation, or continuum mode, in the inelastic neutron scattering (INS) spectrum^6–8^.

Meanwhile, the community recently started to pay attention to the chemical disorder effects on quantum magnetism. For example, some recent studies proposed that the randomness in a quantum magnet can induce spin-singlet dimers of varying strengths in a spatially random manner, which can account for the continuum mode due to its widely distributed binding energy. Indeed, the disorder is unavoidable in most of the studied gapless QSL candidates. For instance, the kagome lattice herbertsmithite ZnCu_3_(OH)_6_Cl_2_ has Zn^2+^/Cu^2+^ site mixture^9^ (whether there is a small gap in this QSL candidate is still under debate); the LiZn_2_Mo_3_O_8_ with breathing kagome lattice has Li^+^/Zn^2+^site mixture^10^; the Ca_10_Cr_7_O_28_ with bilayer kagome lattice has disorder among the two different Cr^3+^positions^11,12^, and the H_3_LiIr_2_O_6_ with honeycomb lattice has mobile Hydrogen ions^13^. Therefore, this so-called random-singlet (RS) or valence bond glass state^14–21^ seriously prompts re-consideration of the intrinsic magnetic ground state of them: whether they are true gapless QSL or just spin liquid-like?

YbMgGaO_4_ (YMGO), a TAF with the effective spin-1/2 Yb^3+^ ions^22,23^, is at the center of this controversy. On one hand, the observed continuum mode^7,8,24,25^, the *C*_p_ ~ _*T*_^0.7^ behavior for the specific heat^22^, the temperature-independent plateau for the Muon spin relaxation (MuSR) rate^26^, and the saturated DC susceptibility below 0.1–0.2 K^27^ all suggest a gapless QSL state. Its origin has been interpreted as a U(1) QSL state with spinon Fermi surface^8,25,27,28^, or a resonant valence bond-like state^24^, or a *J*_1_–*J*_2_ exchange interactions resulted in QSL^7,29^. On the other hand, no residual *κ*_0_/*T* term on the thermal conductivity has been observed^30^. It needs to be emphasized that since the itinerant spin excitations, spinons, carry entropy^5,31–33^, a nonzero residual linear *κ*_0_/*T* term at ultralow temperature for thermal conductivity should exist. It is a well-recognized signature for spinon. The absence of the *κ*_0_/*T* term in YMGO makes it difficult to reconcile with spinon physics. The frequency-dependent AC susceptibility peak^34^ further suggests that the disordered occupancy of the inter-triangular layers by Mg^2+^and Ga^3+^ions^23,35^ lead to a glassy ground state. By including this disorder, other types of ground states have been proposed, such as the mimicry of a spin liquid^36,37^, RS led spin-liquid-like behavior^16,38^, and randomness induced spin-liquid-like phase in *J*_1_–*J*_2_ model^39^.

In this paper, with the motivation to settle down this dispute, which is certainly significant for QSL studies, we performed thermal conductivity, specific heat, magnetic torque, DC magnetization, and AC susceptibility measurements on high-quality YMGO single crystals at ultralow temperatures with two crystallographic axes and detailed field scans to study its intrinsic magnetic ground state. We observed a residual *κ*_0_/*T* term and series of quantum spin state transitions (QSSTs) in the zero-temperature limit, which strongly suggests that a QSL state with itinerant excitations and quantum spin fluctuations survives disorder in YMGO.

## Results and discussions

### Thermal conductivity

**Fig. 1:**
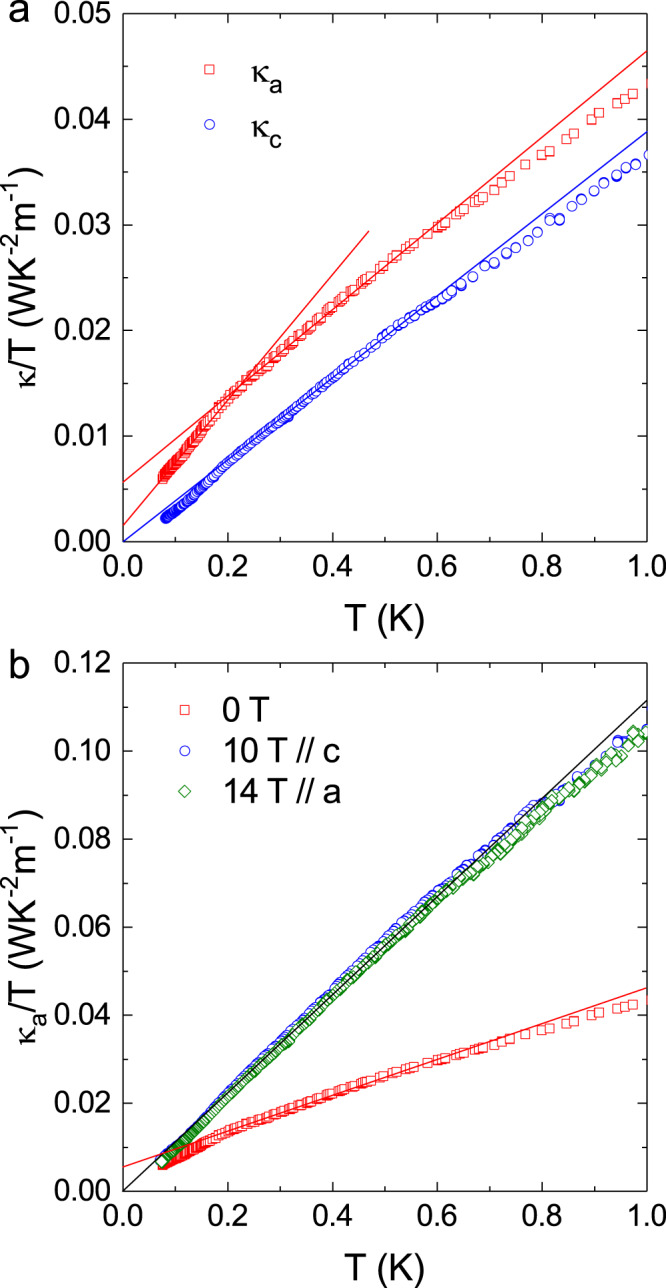
Ultralow-temperature thermal conductivity of YbMgGaO4 single crystals. **a** Data measured along the *a*-axis (*κ*_a_) and the *c-*axis (*κ*_c_), plotted as *κ*/*T* vs. *T*^2^. The solid lines are some linear fitting results. The *κ*_c_ display a *T*^2^ behavior at *T* < 600 mK with zero intercepts at *T* = 0. The *κ*_a_ behave as *T*^2^ at 200 mK < *T* < 600 mK with a residual thermal conductivity of *κ*_0_/*T* = 0.0058 W K^−2^ m^−1^ and a smaller *κ*_0_/*T* = 0.0016 W K^−2^ m^−1^ at *T* < 200 mK. **b** The *a*-axis thermal conductivity in zero field and in 10 T (14 T) magnetic field applied along the *a*- (the *c-*) axis. The high-field *κ*_c_ data display a *T*^2^ behavior at *T* < 700 mK with *κ*_0_/*T* = 0.

Figure 1a shows the ultralow-temperature thermal conductivity (*κ*) of YMGO measured along either the *a* or *c*-axis, plotted with *κ*/*T* vs. *T*. Apparently, the *κ*_a_ displays a *T*^2^ behavior in a rather broad temperature range (200–600 mK) with a slope change at *T* < 200 mK. As shown in Fig. 1a, the *κ*_c_ also exhibits a *T*^2^ behavior at *T* < 600 mK with *κ*_c_/*T* = 0 for zero-temperature limit. Since YMGO is a two-dimensional spin system with negligibly weak spin interaction along the *c*-axis, the *κ*_c_ should represent a pure phonon heat transport of this material. This *T*^2^ behavior for phonon heat transport is further verified by the high-magnetic-field data of *κ*_a_. As shown in Fig. 1a, b, with applying 14 T (or 10 T) field along the *a*-axis (or the *c-*axis), the *κ*_a_ displays a good *T*^2^ behavior at *T* < 700 mK accompanied with *κ*_a_/*T* = 0 for zero-temperature limit. Since these fields are high enough to polarize all the spins, below the magnon gap, the high-field thermal conductivity should be a purely phononic term without any contribution (carrying heat or scattering phonon) from magnetic excitations. It is therefore reasonable that in high fields there is no residual term of *κ*_a_/*T* at *T* → 0. Based on the above comparisons, it is obvious that in zero field the *κ*_a_ behaves as *T*^2^ at 200 mK < *T* < 600 mK with a residual thermal conductivity of *κ*_0_/*T* = 0.0058 W K^−2^ m^−1^ and thereafter, the slope change leads to a smaller *κ*_0_/*T* = 0.0016 W K^−2^ m^−1^ at *T* < 200 mK. Moreover, the larger *κ*_a_/*T* in high fields indicates that, in zero magnetic field, the phonons are rather strongly scattered by magnetic excitations that are be gapped out in high magnetic fields and suppressed at low temperatures. It firmly suggests the presence of magnetic excitations in the thermal conductivity result at zero magnetic field, and the magnetic excitation certainly does not have a large gap and could be gapless.

It is a bit strange that the phonon thermal conductivity at ultralow temperature has a *T*^2^ behavior. Usually, the phonon thermal conductivity of a high-quality insulating crystal at the boundary scattering limit should have a *T*^3^ temperature dependence. One possible explanation is the surface reflection effect, which could weaken the temperature dependence and gives a *T*-power-law behavior with a power smaller than 3. However, the calculated phonon means free path (see Supplementary Information) is much smaller than the sample width, at least at several hundreds of millikelvins. Therefore, the surface reflection may not be the origin of the *T*^2^ behavior. Here, we would like to leave it as an open question.

The nonzero residual thermal conductivity at zero field immediately implies that YMGO is a QSL with itinerant gapless spin excitations having a long-range algebraic (power-law) temperature dependence. In reality, it is very rare to observe this nonzero *κ*_0_/*T* term in the studied QSL candidates. So far only the organic EtMe_3_Sb[Pd(dmit)_2_]_2_^5,33^ and the inorganic 1T-TaS_2_^40^ exhibit a nonzero *κ*_0_/*T* term, both of which are spin-1/2 TAFs. But it is also notable that some recent studies reported a zero *κ*_0_/*T* term in these two materials^41,42^. By the following ref. ^5^’s method, we estimate the mean free path (*l*_s_) of the spin excitations in YMGO by calculating

1 $$\frac{{\kappa }_{0}}{T}=\frac{\pi {k}_{B}^{2}}{9{{\hslash }}}\frac{{l}_{s}}{{ad}}=\frac{\pi }{9}{\left(\frac{{k}_{B}}{{{\hslash }}}\right)}^{2}\frac{J}{d}{\tau }_{s}$$

Here, *a* (~3.40 Å) and *d* (~25.1 Å) are the in-plane and out-of-plane lattice constants, respectively. From the observed *κ*_0_/*T* = 0.0058 W K^−2^ m^−1^, the *l*_s_ is obtained as 78.4 Å, indicating that the excitations are mobile to a distance 23 times as long as the inter-spin distance without being scattered. In comparison, the *κ*_0_/*T* value of YMGO is ~30 times smaller than that of EtMe_3_Sb[Pd(dmit)_2_]_2_ (~0.19 W K^−2^ m^−1^), and ~10 times smaller than that of 1T-TaS_2_ (~0.05 W K^−2^ m^−1^), which may be attributed to two possible reasons. First, the spinon velocity is much smaller in YMGO due to the small *J* value. Second, the *l*_s_ is much shorter in YMGO than that in EtMe_3_Sb[Pd(dmit)_2_]_2_ (~1.20 μm)^5^, which is related to stronger scattering between phonon and spinon. Note that although 1T-TaS_2_ has a very large *J* value^40^, its *l*_s_ (~50 Å) is also not so long, which may be due to the spin–phonon scattering.

Accordingly, the smaller *κ*_0_/*T* = 0.0016 W K^−2^ m^−1^ below 200 mK leads to a *l*_s_ ~21.6 Å, which still covers 6 times of the inter-spin distance. While the exact origin for this slope change (or reduction of the *κ*_0_/*T*) is not clear, we suspect it should be related to the effect of disorder. Here, 200 mK is comparable to the AC susceptibility peak position observed at 80 mK. This result strongly suggests that despite the heavily chemical disorder on the Mg/Ga sites, the itinerant spin excitations of YMGO survive when approaching zero temperature.

It is necessary to point out that in an earlier study, a *T*^2^ behavior of *κ* measured in the *ab* plane at *T* < 300 mK with a negative intercept at *T* = 0 was observed and was interpreted as the non-existence of magnetic heat transport^30^. However, if one compares those data with our *κ*_a_ data, it can be found that those data also exhibit a slope change at ~300 mK; the data above 300 mK exhibit a *T*^2^ behavior with a nonzero *κ*_0_/*T* = 0.0018 W K^−2^ m^−1^. In the present work, first, we measured the thermal conductivity along both the *a-* and *c*-axis. As discussed above, the comparison between the *κ*_a_ and *κ*_c_ clearly show the existence of *κ*_0_*/T* term for *κ*_a_. Second, the phonon mean free path of our sample is much larger than that of ref. ^30^ (see Fig. S2 in Supplementary Information). Therefore, it is very clear that our samples display better thermal conductivity, indicating higher sample quality, and should exhibit more intrinsic physical properties of YMGO. Nevertheless, the ultralow-temperature thermal conductivity data from different groups all indicate that at temperatures above 200 or 300 mK, there are itinerant spin excitations associated with the QSL state, while the disorder effect suppresses the transport of spin excitations at lower temperatures.

**Fig. 2:**
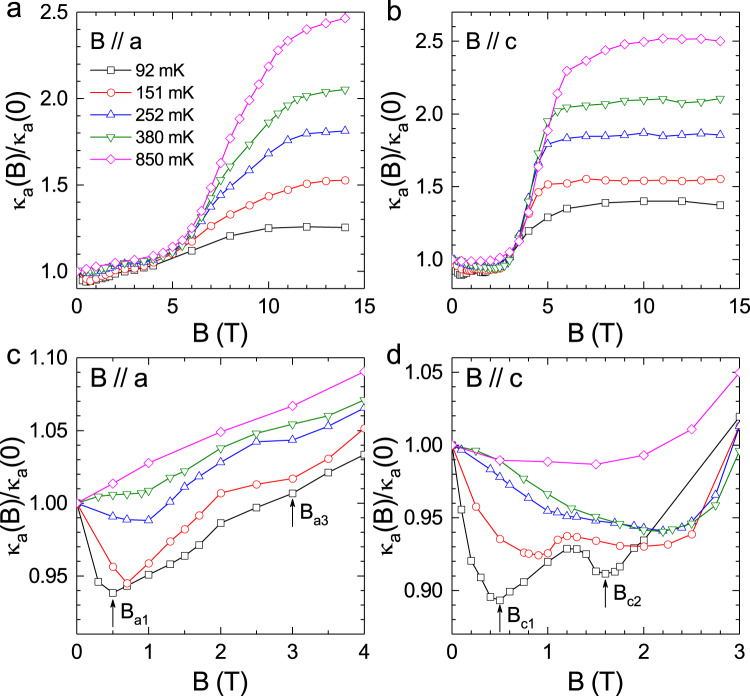
Magnetic field dependence of the a-axis thermal conductivity of YbMgGaO4. **a**, **b** Magnetic field dependence of *κ*_a_ at various temperatures with field-applied along either the *a-*axis or the *c-*axis. **c**, **d** A magnified view of *κ*_a_(*B*) data at low fields. At 92 mK and with *B* // *a*, there is a minimum at 0.5 T and a slope change at 3 T, indicated by arrows. While at 92 mK and with *B* // *c*, there are two minima at 0.5 and 1.6 T. With increasing temperature, these anomalous minima become weaker and disappear above 850 mK.

Figure 2 shows the magnetic-field dependence of *κ*_a_ at various temperatures and with *B* // *a* or *B* // *c*. First, the *κ*_a_ is significantly enhanced at high magnetic fields, indicating the existence of magnetic scattering of phonons that can be smeared out in high fields. Second, the *κ*_a_ exhibits several structures at the low-field region. For *B* // *a*, the *κ*_a_ isotherm measured at 92 mK shows a minimum at *B*_a1_ = 0.5 T and another anomaly at *B*_a3_ ~ 3 T. For *B* // *c*, two more obvious minima are observed for the 92 mK data at *B*_c1_ = 0.5 T and *B*_c2_ = 1.6 T, respectively. These structures under magnetic fields of both directions disappear gradually with increasing temperature.

### Specific heat

**Fig. 3:**
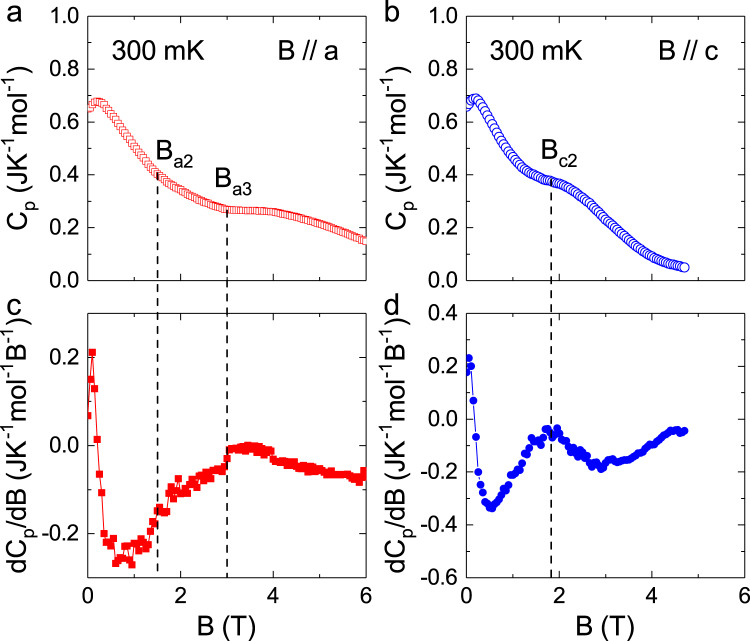
Low-temperature specific heat of YbMgGaO4. **a**, **b** The magnetic-field dependence of specific heat, *C*_p_, measured at 300 mK and with *B* // *a* or *B* //*c*. **c**, **d** The derivative (d*C*_p_/d*B*) for *B* // *a* or *B* //*c*. The dashed lines are the guide for eyes and indicate the anomalies associated with some transition fields *B*_a2_, *B*_a3_, and *B*_c2_.

Figure 3 shows the magnetic-field dependence of specific heat (*C*_p_) at 300 mK with *B* // *a* or *B* // *c*. For *B* // *a*, the *C*_p_ isotherm shows an obvious slope change at *B*_a3_ ~ 3.0 T. Its derivative additionally shows another change around *B*_a2_ = 1.5 T, which corresponds to a weaker slope change of *C*_p_. For *B* // *c*, one clear slope change occurs at *B*_c2_ = 1.7 T, which is also highlighted as a peak on its derivative. These features, both shown by the *κ* and *C*_p_, pointing to some field-induced crossovers or transitions, or some special field-dependent magnetic scatterings.

### Magnetic torque

**Fig. 4:**
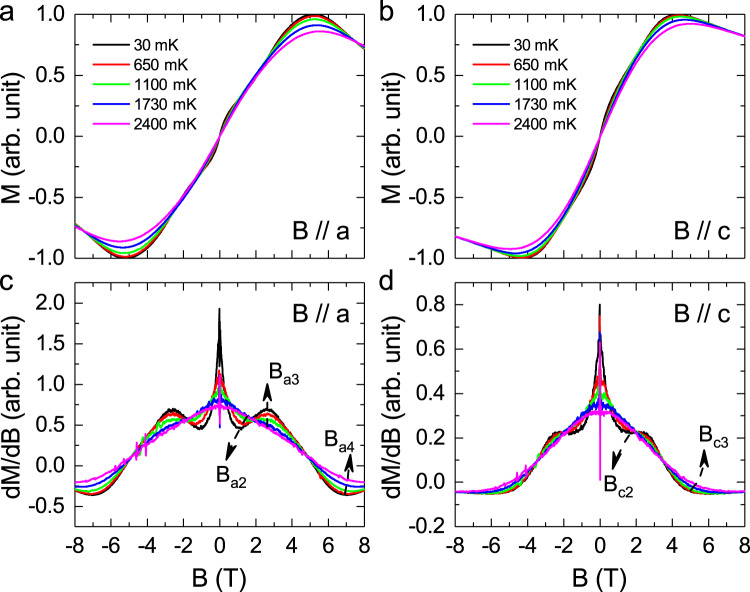
Ultralow-temperature magnetization of YbMgGaO4. **a**, **b** The calculated magnetization (*M*) from the measured magnetic torque as torque/*B* with field-applied along either the *a-*axis or the *c-*axis (see Supplementary Fig. S1). **c**, **d** The derivative (d*M*/d*B*) for *B* // *a*- or *c-*axis. At 30 mK, the derivative shows two anomalies at *B*_a2_ and *B*_a3_ for *B* // *a*, which are related to 1/3*M*_s_ and $\sqrt{3}/3$*M*_s_, respectively. Meanwhile, only one anomaly at *B*_c2_ was observed from the derivative for *B* //*c*, which is related to 1/2*M*_s_.

To further investigate the possible transitions, magnetic torque (*τ*) measurements were performed. Figure 4a, b shows the calculated *τ*/*B* with applied field along the *a*- or *c-*axis. At 30 mK, the data shows a weak hump around 1–2 T for both directions. In principle, torque magnetometry measures magnetic anisotropy. Accordingly, the *d*(*τ*/*B*)/d*B* for *B* // *a* (Fig. 4c) clearly shows a valley at *B*_a2_ = 1.2 T, a peak at *B*_a3_ = 2.7 T, and another valley at *B*_a4_ = 7.0 T. With increasing temperature, the lower field valley, and peak both become weak and disappear with *T* ≥ 1.5 K. For *B* // *c*, the *d*(*τ*/*B*)/d*B* (Fig. 4d) does not show a peak but a flat regime starting around *B*_c2_ = 1.7 T, and a valley at *B*_c3_ = 5.0 T. The *B*_a2_*, B*_a3_, and *B*_c2_ observed here are closely corresponded to the anomaly observed from the *κ*(*B*) and *C*_p_(*B*) data. This further confirms the existence of field-induced magnetic crossovers or transitions. It is noticed that the magnetic torque was also measured for YMGO at 350 mK by Steinhardt et al.^43^ Its derivative shows broad maxima near 3.5 T for *B* // *c* and 5.5 T for *B* // *a*, which is different from our results obtained at 30 mK while approaching zero temperature.

It is noticed that a recent study reported the DC magnetization measured at 500 mK with *B* // *ab* plane^44^ or *B* // *c*. We also measured the 500 mK magnetization with *B* // *a* or *B* // *c*. Our results are the same as the reported ones, as shown in Fig. S4 in Supplementary Information. For *B* // *a*, the saturation field *B*_s_ is around 7.0 T with saturation moment *M*_s_ = 1.45 *μ*_B_. For *B* // *c*, *B*_s_ = 5.0 T and *M*_s_ = 1.8 *μ*_B_. Therefore, the *B*_a4_ and *B*_c4_ represent the saturation fields. It is also noted that the magnetization at *B*_a2_ = 1.5 T, *B*_a3_ = 3.0 T, and *B*_c2_  = 1.7 T is around 1/3, $\sqrt{3}/3$, and 1/2 of the saturation value, respectively, with the assumption that the critical field values are similar between 300 and 500 mK. In ref. ^44^, they further measured magnetization at 40 mK with *B* // *c*. Its derivative shows a flat regime around 1.8 T, which is related to 1/2*M*_s_. This result is consistent with our observation here. For their data measured at 500 mK with *B* // *ab* plane, its derivative shows a flat regime around 3.5 T, which again was ascribed to 1/2*M*_s_. This explanation is different from our observation for *B* // *a* case, in which both phase boundaries at 1/3*M*_s_ and $\sqrt{3}/3$*M*_s_ were observed by the combination of *κ*(*B*), *C*_p_(*B*), and *τ*(*B*) data. One reason for this discrepancy could be the fact that the DC magnetization was measured at 500 mK, a relative high temperature. As shown in our data, Fig. 4c, the anomalies on the d(*τ*/*B*)/d*B* curve at 30 mK, an ultralow temperature, become weak or flattened pretty quickly with increasing temperature. The *C*_p_(*B*) data at 200 mK was also reported in ref. ^44^. However, its *C*_p_(*B*) data with *B* // *ab* plane shows no distinct feature around 3.0 T, which our *B* // *a* data clearly shows. The slope change around 1.5 T, as we observed, also has not been discussed in ref. ^44^. Meanwhile, we also noticed that an early study on the DC magnetization measured on powder sample at 500 mK reported two critical fields at 1.6 and 2.8 T^22^, which were suggested as the phase boundaries of an up–up–down (UUD) phase. This study supports our observation with *B* // *a*.

### Phase diagram

**Fig. 5:**
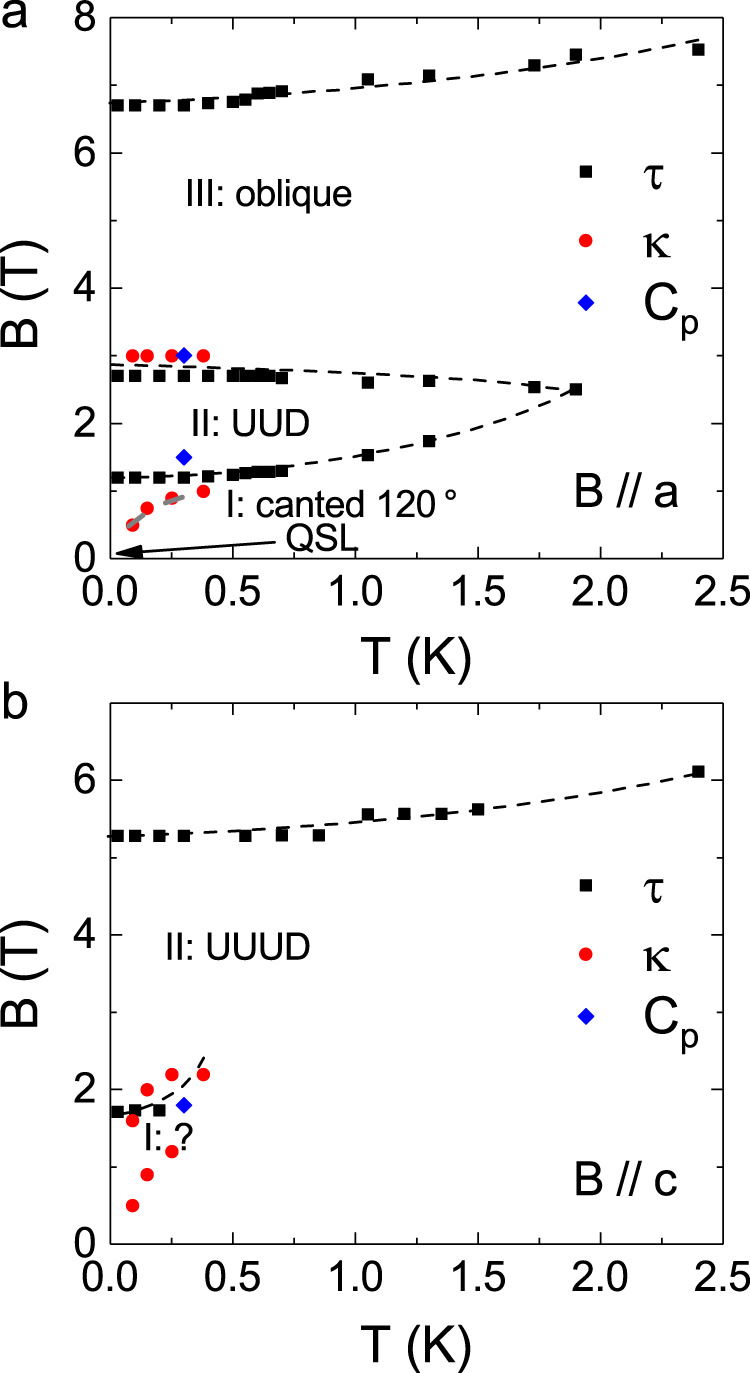
Magnetic phase diagrams of YbMgGaO4. **a** For *B* // *a*. **b** For *B* // *c*. The data points are obtained from the magnetic torque (*τ*) and field dependence of thermal conductivity (*κ*) measurements. The dashed lines are phase boundaries. For *B* // *a*, there are three phases (I: thee canted 120° spin structure, II: the UUD phase, and III: the oblique phase) in the low-temperature and low-field region. Whereas, there are two phases (I: unknown phase and II: UUUD phase) at low temperature and low field for *B* // *c*. The dashed lines are the guide to the eyes.

Two magnetic phase diagrams are constructed by using these critical fields, as shown in Fig. 5. Since no anomaly was observed on *τ*/*B* around 0.2–0.5 T, the *B*_a1_ and *B*_c1_ are unlikely related to spin-state transitions. Accordingly, besides the paramagnetic phase at high temperatures, QSL at low temperature and zero field, and fully polarized phase at high fields, with increasing field, there are three phases for *B* // *a* (Fig. 5a) and two phases for *B* // *c* (Fig. 5b).

For a spin-1/2 TAF with 120° spin ordering and easy-plane anisotropy, the strong quantum spin fluctuations can stabilize a so-called UUD phase within a certain magnetic field regime applied in the triangular plane while approaching zero temperature^45–49^, which has been observed in Ba_3_CoSb_2_O_9_^50–54^, a TAF with effective spin-1/2 Co^2+^ ions. This UUD phase exhibits a magnetization plateau with 1/3 saturated moment (*M*_s_). Recently, even in QSL candidates *A*Yb*Ch*_2_ (*A* = Na and Cs, *Ch* = O, S, Se), one TAF family with effective spin-1/2 Yb^3+^ ions and easy-plane anisotropy, the UUD phase has been proposed under applied fields^55–58^. Experimentally, more complex QSSTs have been observed for Ba_3_CoSb_2_O_9_^59–65^. For example, with increasing field along the *ab* plane, its 120° spin structure at zero field is followed by a canted 120° spin structure; the UUD phase; a coplanar 2:1 canted oblique phase with one spin in the 120° spin structure rotated to be parallel with another spin, which gives $\sqrt{3}/3$*M*_s_; and another coplanar phase before entering the fully polarized state.

Since YMGO also has the easy-plane anisotropy^7,8,29^, its field-induced anomaly at ultralow temperatures could also be related to these QSSTs. By comparing YMGO’s phase diagram with *B* // *a* to that of Ba_3_CoSb_2_O_9_, we propose a phase I as the canted 120° spin structure, phase II as the UUD phase, and phase III as the oblique phase. The observation of the QSSTs strongly supports the existence of quantum spin fluctuations approaching zero temperature in YMGO, which clearly differentiate it from a conventional spin glass state. It needs to be pointed out is that while the phase boundaries of the UUD phase were observed, the 1/3*M*_s_ plateau does not exist for YMGO, which could be due to the disorder effect. In another TAF Rb_1−*x*_K_*x*_Fe(MoO_4_)_2_^66^, the disorder introduced by the K-doping also weakens the magnetization plateau feature related to the UUD phase.

It is surprising to see that the phase boundary between phase I and II for *B* // *c* case is related to 1/2 *M*_s_. As learned from Ba_3_CoSb_2_O_9_, while for *B* // *c*, the 120° spin structure will be followed by an umbrella spin structure and an oblique phase, between which the phase boundary is related to $\sqrt{3}/3$*M*_s_. Meanwhile, Ye and Chubukov calculated the phase diagram of a 2D isotropic triangular Heisenberg antiferromagnet in a magnetic field and predicted a novel up–up–up–down (UUUD) spin sate with a 1/2*M*_s_ magnetization plateau for *J*_2_/*J*_1_ > 0.125 (*J*_1_: nearest-neighbor exchange interaction, *J*_2_: next nearest-neighbor exchange interaction)^67^. The reported *J*_2_/*J*_1_ values by simulating the spin excitations obtained from INS data^7^ and terahertz spectroscopy data^29^ are 0.22 and 0.18, respectively. Therefore, we tend to assign phase II as the UUUD phase, while the nature of Phase I is not clear at this stage. Again, the chemical disorder could be the reason for the disappearance of the 1/2*M*_s_ plateau.

In the case that the minimum observed at 0.5 T for *κ*(*B*) belong to some special magnetic scatterings, the scenario of a spinon Fermi surface QSL, that supports gapless magnetic excitations discussed above, may give a possible understanding from the conventional wisdom of Kohn anomaly. Since the charge degrees of freedom in YMGO are frozen out, only Zeeman coupling can be included as the magnetic field is applied. In the weak-field regime, the field does not destroy the QSL ground state and the spinon remains to be a valid description of the magnetic excitation^28^. The magnetic fields would modify the spinon Fermi surface, and the Fermi surfaces of spin-up and spin-down spinons expand and shrink with increasing field, respectively. Just like the electron-phonon coupling^68^, the spin–lattice coupling may enhance the phonon scatterings with modified spinon Fermi surfaces, resulting in the thermal conductivity modulation. In the high-temperature regime, the QSL breaks down and the structures disappear as in the experiment.

### AC susceptibility

**Fig. 6:**
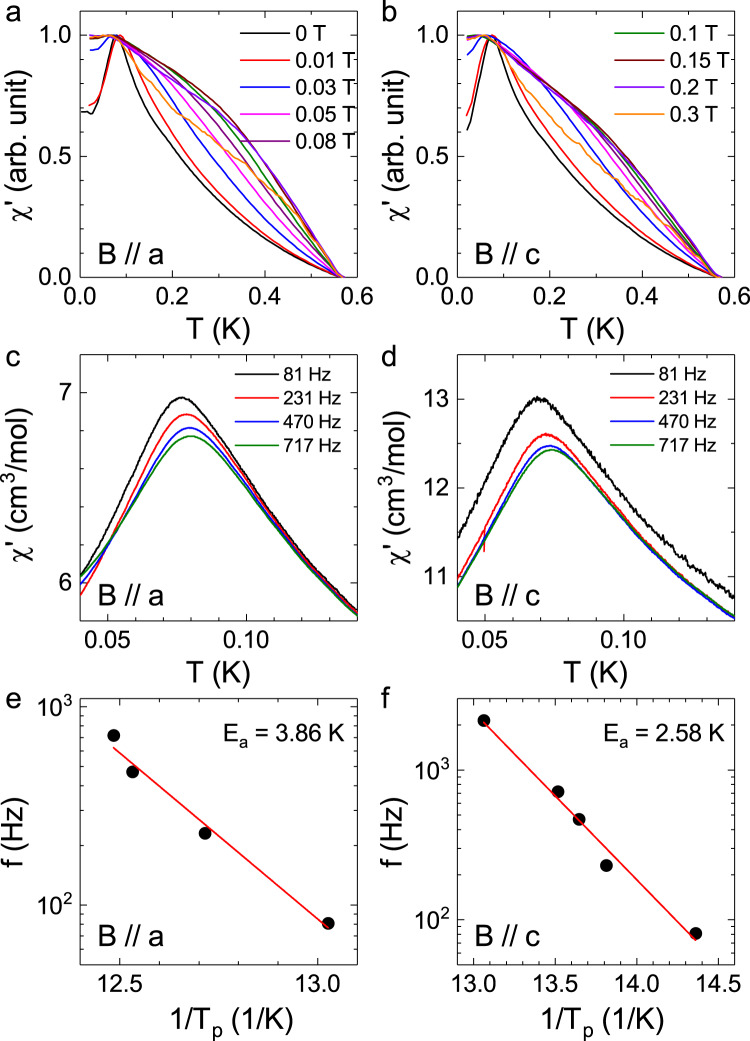
AC susceptibility of YbMgGaO4. **a**, **b** Temperature dependence of the real part of the AC susceptibility, *χ*′, measured with different DC magnetic fields along either the *a*-axis or the *c-*axis. Here, the maximum value of each data was scaled to 1 to clearly show the DC magnetic field effects on AC susceptibility. **c**, **d** The Frequency dependence of the *χ*′ peak. Here, the absolute value of the AC susceptibility was obtained by rescaling it to the DC susceptibility (see Supplementary Information). **e**, **f** Arrhenius law fit of the *χ*′ peak position, *T*_0_. The applied AC excitation field (~1 Oe) is along either the *a-*axis for (**c**, **e**) or the *c*-axis for (**d**, **f**).

Finally, we revisited the AC susceptibility (*χ*′) to check the possibility for a spin glass state in YMGO, although the observation of the residual *κ*_0_/*T* and QSSTs clearly disputes this scenario. Compared to the reported *χ*′ data performed at zero DC magnetic field and without anisotropic information, our *χ*′ data was measured with AC field both along the *a-* and *c*-axis, and with applied DC field. Figure 6a, b shows the *χ*′(*T*) measured with applied DC field along either the *a-* or *c-*axis. With *B* = 0 T, the *χ*′ shows a peak around 80 mK, which is lower than the reported data exhibiting a peak around 100 mK^34^. With increasing *B*, the intensity of *χ*′ below 80 mK increases and eventually becomes flat or saturated with *B* ≥ 0.05 T. Figure 6c, d shows the frequency dependence of the peak position with the AC excitation field either along the *a-* or *c-*axis. For both of them, the peak’s position (*T*_0_) shifts to higher temperatures with increasing frequency. As shown in Fig. 6e, f, its frequency dependence can be fit to an Arrhenius law *f* = *f*_0_ exp[−*E*/(*k*_B_*T*_0_)], which yields an activation energy *E*_*a*_ = 3.8(6) K and *E*_*c*_ = 2.5(8) K for the excitation field along the *a-* and *c-*axis, respectively. While the frequency-dependent peak of *χ*′ normally represents a spin glass transition as discussed for YMGO and YbZnGaO_4_^34^, the saturation of the *χ*′ below this peak position under a small DC field and the anisotropic activation energy both indicate that it should not be treated as a conventional spin glass^69^.

Meanwhile, the recent DC susceptibility measurements with *B* = 0.01–0.05 T for YMGO^70^ also revealed a saturation below 100–200 mK, which has been suggested as a signature for the presence of gapless spin excitations. Since the temperature dependence of the AC and DC susceptibility should behave similarly, the saturation we observed for *χ*′ could be due to the same origin. In fact, the reported INS measurement suggests that at most 16(3)% of the total spectral weight is elastic^7^. Correspondingly, the inelastic contribution (~84(3)%) is large compared with the 66% expected for a spin-1/2 glass^71^. Moreover, the DC susceptibility shows no divergence between the zero-field cooling and field cooling data, the *μ*SR relaxation rate shows a plateau below 400 mK^26^, and the magnetic entropy has been already released by more than 99% at 80 mK from the specific heat measurement^22^. All these facts again rule out a frozen or glassy state for YMGO. This AC susceptibility peak could originate from the free impurity spins inside or attached to the system.

## Conclusion

In summary, the observation of a residual *κ*_0_/*T* term and QSSTs strongly supports a QSL state with itinerant spin excitations and quantum spin fluctuations approaching zero temperature in YMGO, although its chemical disorder reduces the mean free path of the excitations and smears out the 1/3*M*_s_ plateau related to the UUD phase. This survival of QSL state in YMGO is surprising since the Mg/Ga site disorder is supposed to introduce random distribution of exchange interactions and therefore lead to an RS state or even a glassy state and any field-induced transitions should be expected to smear out completely. Therefore, the chemical disorder in YMGO must play a more complex role in the exchange interactions. Future studies on the local structure of YMGO to learn how exactly the Mg/Ga disorder affects the Yb–O local environment and the correlated exchange interactions are highly desirable to understand this survival of QSL in YMGO through chemical disorder.

## Methods

### Sample preparation and characterization

High-quality YMGO single crystals were grown by using the optical floating-zone technique^8^. By using the X-ray Laue photograph, the crystals were cut precisely along the crystallographic axes for the magnetic and thermal conductivity measurements.

### Thermal conductivity measurements

Thermal conductivity was measured by using a “one heater, two thermometers” technique in a ^3^He/^4^He dilution refrigerator at 70 mK–1 K, equipped with a 14 T superconducting magnet^72,73^. The sample was cut precisely along the crystallographic axes with the longest and the shortest dimensions are along the *a*- or the *c*-axis. The magnetic fields were applied along either the *a-* or *c-*axis. Gold paint was used to make four contacts on each sample. The RuO_2_ chip resistors were used as heaters and thermometers and are connected to the gold contacts by using gold wires and silver epoxy. The RuO_2_ thermometers were pre-calibrated by using a RuO_2_ reference sensor (Lakeshore Cryotronics) mounted at the mixture chamber (the superconducting magnet was equipped with a cancellation coil at the height of the mixture chamber).

### AC susceptibility measurements

The ac susceptibility was measured using the conventional mutual inductance technique (with a combination of ac current source and a lock-in amplifier) at SCM1 dilution fridge magnet of the National High Magnetic Field Laboratory, Tallahassee. The typical AC field strength is 1.1–1.6 Oe.

### Torque measurements

Torque magnetometry was performed at the University of Michigan using a self-built capacitive cantilever setup mounted inside a dilution refrigerator^74–76^. The sample was mounted on a thin BeCu cantilever. To infer the magnetic torque $\tau$, the cantilever deflection is measured by tracking the change of the capacitance between the cantilever and a gold thin film underneath. The report magnetization is the effective torque magnetization *M* = *τ*/*B*.

### Specific heat measurements

The specific heat was measured with a Quantum Design physical property measurements system, equipped with a dilution refrigerator insert.

### DC magnetization measurements

The DC magnetization curves were measured with a Quantum Design SQUID-VSM, equipped with a ^3^He refrigerator insert.

## Supplementary information

Supplementary Information

## Data Availability

The data that support the findings of this study are available from the corresponding authors upon reasonable request.
