# Supplementary material for: Survival of itinerant excitations and quantum spin state transitions in YbMgGaO4 with chemical disorder
Source: Nat Commun. 2021 Aug 16;12:4949. doi: 10.1038/s41467-021-25247-6 (PMC8367942; doi:10.1038/s41467-021-25247-6)
Supplement: Supplementary file 1 — Supplementary Information [file 41467_2021_25247_MOESM1_ESM.docx]

**Supplementary Information for**

***“Survival of itinerant excitations and quantum spin state transitions in YbMgGaO_4_ with chemical disorder”***

X. Rao^1,10^, G. Hussain^1,10^, Q. Huang^2,10^, W. J. Chu^1^, N. Li^1^, X. Zhao^3^, Z. Dun^2^, E. S. Choi^4^, T. Asaba^5^, L. Chen^5^, L. Li^5^, X. Y. Yue^6^, N. N. Wang^7^, J.-G. Cheng^7^, Y. H. Gao^8^, Y. Shen^8^, J. Zhao^8^, G. Chen^9,8★^, H. D. Zhou^2★^, and X. F. Sun^1,6★^

^1^Hefei National Laboratory for Physical Sciences at Microscale, Department of Physics, and Key Laboratory of Strongly-Coupled Quantum Matter Physics (CAS), University of Science and Technology of China, Hefei, Anhui 230026, People’s Republic of China

^2^Department of Physics and Astronomy, University of Tennessee, Knoxville, Tennessee 37996-1200, USA

^3^School of Physical Sciences, University of Science and Technology of China, Hefei, Anhui 230026, People’s Republic of China

^4^National High Magnetic Field Laboratory, Florida State University, Tallahassee, FL 32310-3706, USA

^5^Department of Physics, University of Michigan, Ann Arbor, Michigan 48109, USA

^6^Institute of Physical Science and Information Technology, Anhui University, Hefei, Anhui 230601, People’s Republic of China

^7^Beijing National Laboratory for Condensed Matter Physics and Institute of Physics, Chinese Academy of Sciences, Beijing 100190, People’s Republic of China

^8^State Key Laboratory of Surface Physics and Department of Physics, Fudan University, Shanghai 200433, People’s Republic of China

^9^Department of Physics and HKU-UCAS Joint Institute for Theoretical and Computational Physics at Hong Kong, The University of Hong Kong, Hong Kong, China

^10^These authors contributed equally: X. Rao, G. Hussain, Q. Huang

^★^email: gangchen.physics@gmail.com; hzhou10@utk.edu; xfsun@ustc.edu.cn

***Torque data*:**

**Supplementary Figure S1** Field dependence of the magnetic torque of YbMgGaO_4_ at different temperatures. **a,** for *B* // *a*. **b,** for *B* // *c*.

***Phonon mean free path:***

**Supplementary Figure S2** Temperature dependence of the phonon mean free path *l*_ph_ divided by the averaged sample width *W*, calculated from our *κ*_a_ sample with 10 T // *c* and the sample in Ref. S4 with 9 T // *c*.

It is notable that since all the thermal conductivity data (including both *κ*_a_ and *κ*_c_, zero field and high field) display a temperature dependence close to *T*^2^. Usually, the phonon thermal conductivity of a high-quality insulating crystal at boundary scattering limit should have a *T*^3^ temperature dependence. One possible reason is related to the surface reflection effects, which could result in phonon mean free path long than the sample size and a *T*-power-law behavior with a power smaller than 3. Therefore, it is useful to calculate the phonon mean free path from our thermal conductivity data. First, we analyzed the specific heat data of LuMgGaO_4_, which are purely phononic and can be a reference of the phonon specific heat of YbMgGaO_4_. We got the raw data (at 0.26−30 K) from Ref. S1 and fitted the data by using the low-temperature expansion of the Debye function, *C* = *βT*^3^ + *β*_5_*T*^5^ + *β*_7_*T*^7^ (see Refs. S2 and S3). The fitting parameters are *β* = 5.19×10^-4^ J/K^4^mol, *β*_5_ = -3.24×10^-7^ J/K^6^mol, and *β*_7_ = 6.60×10^-11^ J/K^8^mol. The phononic thermal conductivity can be expressed by the kinetic formula *κ*_ph_ = 1/3*Cv*_ph_*l*_ph_, where *C* = *βT*^3^ is phonon specific heat at low temperatures, *v*_ph_ is the average velocity, and *l*_ph_ is the mean free path of phonon. Here *β* = 5.19×10^-4^ J/K^4^mol is obtained from the above specific-heat data and *v*_ph_ = 2070 m/s can be calculated from *β* (see Ref. S3). Then, we can calculate *l*_ph_ from the *κ*_a_(*T*) data at 10 T field (// *c*) and compare it with the averaged sample width *W* = 2(*A*/*π*)^1/2^ = 0.375 mm (for the *κ*_a_ sample), where *A* is the area of cross section. For comparison, we also analyzed the data in Ref. S4 (in-plane thermal conductivity with 9 T // *c*) and calculated the mean free path of phonons. In Figure S2, we plot the temperature dependence of *l*_ph_/*W* of our *κ*_a_ sample with 10 T // *c* and the sample in Ref. S4 with 9 T // *c*. Apparently, our samples display better thermal conductivity, indicating higher sample quality, and should exhibit more intrinsic physical properties of YMGO.

Although the phonon mean free path keeps increasing with decreasing temperature, it is still smaller than the averaged sample width at the lowest temperatures. This might be related to some uncertainties in the above fitting and calculations, such as the determination of the *β* coefficient, the slight difference in phonon specific heat between YbMgGaO_4_ and LuMgGaO_4_, etc. However, it is more likely that the phonon mean free path is much smaller than the sample width, at least at several hundreds of millikelvins. Therefore, the surface reflection may not be the origin for the *T*^2^ behavior of thermal conductivity.

Except for the surface reflection effect, it is very hard to understand a *T*^2^ behavior of phonon thermal conductivity at such low temperatures. Usually at subkelvin temperatures, phonons are free from the microscopic scattering by lattice imperfections, like point defects and dislocations, and *κ*_ph_ displays a temperature dependence close to *T*^3^. One may suspect that it is due to the magnetic scattering effect. This scattering is indeed playing a role considering the rather strong magnetic-field dependence of *κ*. However, high magnetic field is believed to suppress the magnetic excitations and smear out the magnetic scattering effect. Thus, it is still a mystery why the high field data, which is purely phononic, display a *T*^2^ behavior. We would like to leave it as an open question.

Nevertheless, since all the data (including both *κ*_a_ and *κ*_c_, zero field and high field) display a temperature dependence close to *T*^2^, it is reasonable to analyze the low temperature data by using the *κ*/*T* vs *T* and to get the intercept values at *T* = 0.

***Magnetic susceptibility scaling***:

**Supplementary Figure S3** The magnetic susceptibility for YbMgGaO_4_ shown with DC data and scaled AC data.

The temperature dependence of the AC susceptibility measured with a small AC field and low frequency should reflect the intrinsic susceptibility behavior of a system, or has the similar temperature trend of the DC susceptibility measured on the same system. Therefore, a AC field of 1 Oe with frequency 231 Hz was used to measure the magnetic susceptibility down to 30 mK. This data was easily matched to the high temperature DC susceptibility data measured with a DC a field at 100 Oe and taken down to 1.8 K with a simple scaling factor. Unit of the DC susceptibility, cm^3^/mol, is used for scaled AC data to maintain continuity. The data in Figures 5c and 5d of the main text with absolute value was obtained by this scaling.

**Figure S4 DC magnetization of YbMgGaO_4_ at 500 mK. a,** for *B* // *a*. **b,** for *B* // *c*. The magnetization at *B*_a2_ = 1.5 T, *B*_a3_ = 3.0 T, and *B*_c2_ = 1.7 T is around 1/3, $\sqrt{3}/3$and 1/2 of the saturation value, respectively. The thick dashed line in **b** indicates the Van Vleck paramagnetic background.

**References**

S1. Li, Y. *et al*. Gapless quantum spin liquid ground state in the two-dimensional spin-1/2 triangular antiferromagnet YbMgGaO_4_. *Sci. Rep.* **5**, 16419 (2015).

S2. Tari, A. *Specific Heat of Matter at Low Temperatures* *Ch. 2* (Imperial College Press, London, 2003).

S3. Zhao, Z. Y. *et al.* Magnetic phase transitions and magnetoelectric coupling of GdFeO_3_ single crystals probed by low-temperature heat transport. *Phys. Rev. B* **83**, 014414 (2011).

S4. Xu, Y. *et al.* Absence of Magnetic Thermal Conductivity in the Quantum Spin-Liquid Candidate YbMgGaO_4_. *Phys. Rev. Lett.* **117**, 267202 (2016).
